# Materialization of single multicomposite nanowire: entrapment of ZnO nanoparticles in polyaniline nanowire

**DOI:** 10.1186/1556-276X-6-393

**Published:** 2011-05-20

**Authors:** Innam Lee, Seong Yong Park, Moon J Kim, Minhee Yun

**Affiliations:** 1 Department of Electrical and Computer Engineering, University of Pittsburgh, Benedum Hall 348, Pittsburgh, PA 15261, USA; 2 Department of Electrical Engineering, University of Texas at Dallas, Richardson, TX 75080, USA

## Abstract

We present materialization of single multicomposite nanowire (SMNW)-entrapped ZnO nanoparticles (NPs) via an electrochemical growth method, which is a newly developed fabrication method to grow a single nanowire between a pair of pre-patterned electrodes. Entrapment of ZnO NPs was controlled via different conditions of SMNW fabrication such as an applied potential and mixture ratio of NPs and aniline solution. The controlled concentration of ZnO NP results in changes in the physical properties of the SMNWs, as shown in transmission electron microscopy images. Furthermore, the electrical conductivity and elasticity of SMNWs show improvement over those of pure polyaniline nanowire. The new nano-multicomposite material showed synergistic effects on mechanical and electrical properties, with logarithmical change and saturation increasing ZnO NP concentration.

## Background

New nano-multicomposite materials have been researched with the goal of producing new materials with vastly improved physical and chemical properties. Nano-multicomposite materials are being developed for use in a broad range of electrical, bio-medical, and mechanical engineering applications and provided excellent electrical conductivity and mechanical strength resulting from reactions between the composites in synthesis [1,2]. The nano-multicomposite materials are typically produced as a mixture of either carbon nanotubes (CNTs), graphene or nanoparticles (NPs) with an organic material such as a conducting polymer [3-6]. These composite materials are intriguing because each individual component's complementary nature in the mixture acts synergistically for improved physical and chemical properties [7-9]. For example, CNT-polymer multicomposites synthesized by in situ polymerization, in thin film and nanowire structures, have shown improved electrical conductivity, photoluminescence, and mechanical strength [10-14]. Likewise, bundled CNT-Polypyrrole (PPy) nanowires fabricated from anodic aluminum oxide (AAO) templates via cyclic voltammetry demonstrate higher electrical conductivity than PPy nanowires [15,16]. In the case of CNT-PPy composites, the end result displays a metallic character, whereas PPy nanowires serve as semiconductors. Other inorganic nanowire-polymer composites of ZnO, RuO2, and Ag with polyaniline (PANI) or PPy demonstrate varying electrical conductivity according to synthesis types (in situ or ex situ polymerization) and as a function of the mix composition [17]. These nano-multicomposite nanowires are fabricated in bundles via various methods such as the AAO template method, electrochemical deposition, and electrospinning [13-17].

Application of nanowires is difficult when utilizing the mass production growth methods mentioned above. First, individually selected nanowires must be extracted from the bundles using various methods, often requiring several non-scalable post-processing steps [8,10]. The selection and alignment of a selected nanowire for implementation in a usable testing device are both a time-consuming and labor-intensive process. Furthermore, these nanowire devices have disadvantages with respect to addressability, uniformity of structure and performance as a result of differences in the required post-processes [15,16].

In order to overcome the limits mentioned above, another method for the fabrication of nanowire devices is being developed which utilizes electrochemical deposition of organic or inorganic materials in pre-patterned nanochannels by applying static current between two electrodes [18]. The electrochemical growth method offers an alternative fabrication with simple equipment requirements to grow a single nanowire. This nanowire fabrication method produces single site-specific nanowire devices with excellent reproducibility, uniformity, and cost efficiency, in addition to requiring fewer post-processing steps [19,20]. For example, single Pd nanowires fabricated using this method have been used in hydrogen sensors with the lowest detection limits (2 ppm) ever recorded by a nanowire device [19]. Similarly, single-PANI nanowires have electrical conductivity which can be controlled by the pH of aniline solution, as well as by a simple post-process such as acetone wetting [20].

In this research, we suggest a newly developed method for fabrication and characterization of single multicomposite nanowires (SMNWs) based on the electrochemical growth method. For this report, single ZnO NPs-entrapped PANI nanowires have been fabricated using the electrochemical growth method, and these show improved physical properties. The growth of SMNWs is similar to that of single conducting polymer nanowires, except that ZnO NPs are attracted to the nanochannel via an electric field applied from the electrodes, while in situ polymerization of PANI occurs simultaneously. ZnO NPs were chosen because of their controllable conductivity, wide-bandgap and optical transparency, all of which make them useful for various applications [21,22]. ZnO NPs are also a good candidate for biosensing materials with their high sensitivity [23]. PANI was the polymer of choice because of its excellent bioaffinity, cost efficiency, environmental stability, and ease of synthesis [24]. Through modulation of ZnO NPs and PANI components, the goals of this research are to create a synergistic compound with tailorable physical characteristics and a new noble material which can be utilized for electric and biosensing applications. We successfully show that the fabricated SMNWs with uniform dimensions and structure demonstrate changes in mechanical strength and electrical conductivity dependent on ZnO NP concentration (1, 2.5, 5, 10, and 20 wt.%). In addition, we show the entrapment of ZnO NP concentration in SMNWs along different growth conditions such as applied electric potential.

## Results

### Fabrication of single multicomposite nanowires

**Figure 1:**
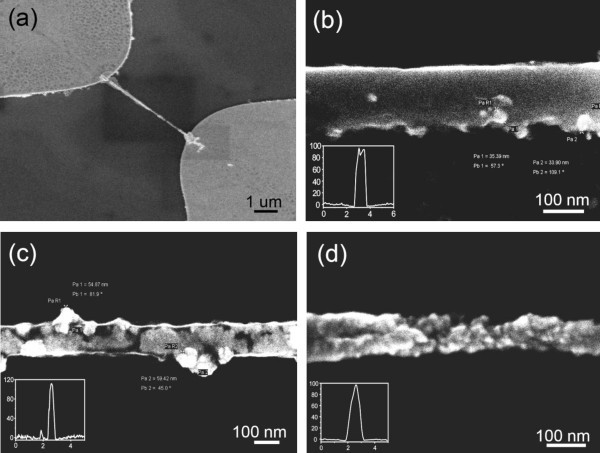
**Fabricated SMNWs with different ZnO NPs concentrations**. (a) Low-resolution SEM image of 5-μ m-long SMNW, entrapped ZnO NPs with uniform structure. **(b) **Highly magnified image of 5 wt.% ZnO NP-entrapped PANI nanowire with 97 nm height and 133 nm width. Diameters of the observed particles are 33.90 and 35.39 nm. **(c) **10 wt.% ZnO NP-entrapped PANI nanowire with 108 nm height and 110 nm width. Diameters of the observed particles are 54.87 and 59.42 nm. **(d) **20 wt.% ZnO NP-entrapped PANI nanowire with 98 nm height and 80 to 110 nm width. It can be seen that the agglomerated particles greatly changes surface morphology of the SMNW with taped shape.

Figure 1 shows SMNWs grown along a 5-μ m-long nanochannel. These SMNWs have a very uniform width of 108 to 133 nm and a height of 97 to 112 nm, measured using both scanning electron microscopy (SEM) and atomic force microscope (AFM). Figure 1a shows the uniform structure of the SMNW is comparable to that of the single PANI nanowire as demonstrated in our previous research [20]. Highly magnified images of the SMNWs for 5, 10, and 20 wt.% of ZnO NP concentrations are shown in Figure 1b, c, d, respectively. In these SEM images, some observed contrast spots on the SMNWs are assumed to be ZnO NPs with a diameter of 30 to 60 nm. This range is consistent with the 20 to 70 nm range listed in the datasheet from Alfa Aesar. The 5 wt.% ZnO NPs SMNW shows a uniform 133 nm width and smooth topography in Figure 1b. The observed particles were 33.90 and 35.39 nm in diameter, respectively (measured with an SEM). The 10 wt.% ZnO NP-entrapped PANI nanowire displays a regular 117 nm width and a greater number of ZnO NPs than the 5 wt.% ZnO NP concentration, as might be expected in Figure 1c. The 20 wt.% ZnO NP-entrapped PANI nanowire displays widths varying from 60 to 100 nm in Figure 1d. Increases in the density of the NPs and change of surface morphology of the SMNWs are clear when comparing Figure 1b, c. In the insets of Figure 1b, c, d, the AFM line scans show height variation from 97 to 112 nm along the nanowire.

**Figure 2:**
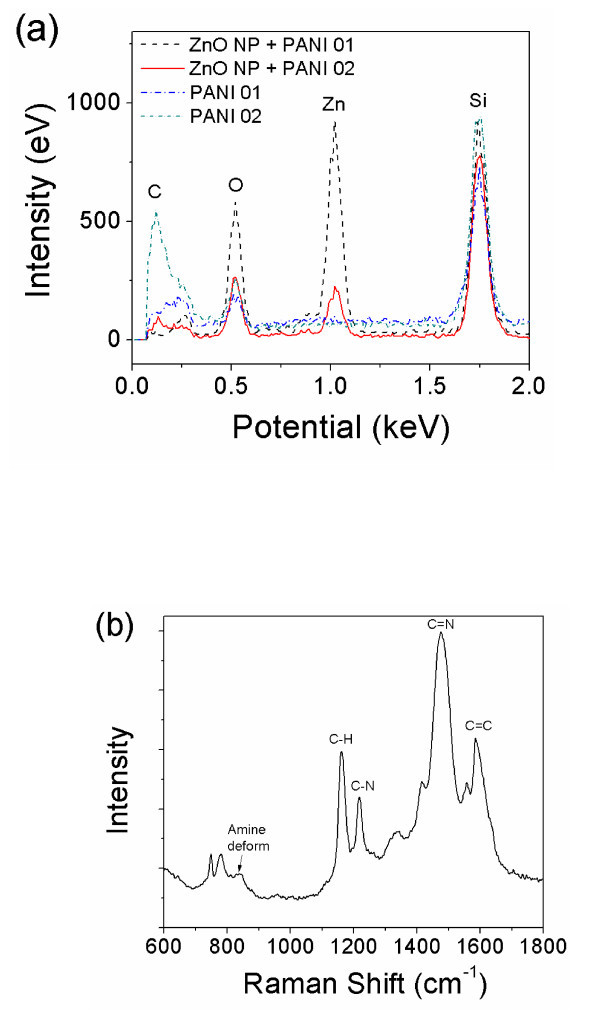
**EDX data for single PANI nanowires and single ZnO NPs-entrapped PANI nanowires**. (a) The SMNWs with ZnO NP show clear peaks at Zn and a higher peak at O resulting from the entrapment. The two different ZnO concentrations (black dashed line: 20 wt.%, red solid line: 5 wt.%) display different intensities of Zn. **(b) **Raman spectrum of fabricated single PANI nanowire.

As the ZnO NP concentration increased, the NPs aggregated tightly in SMNW, and the structure of SMNW was affected. Thus, the ZnO NP concentration clearly affects the morphology of the SMNWs, as demonstrated in the SEM images. To further prove that the contrast spots are indeed ZnO and that our fabrication method works for nano-multicomposite material, we investigated the nanowires composition with energy-dispersive x-ray spectroscopy (EDX) and Raman spectroscopy as shown in Figure 2a, b. EDX data shows different peaks corresponding to the elements C, O, Si, and Zn. C, Si, and O are from the PANI and the SiO_2 _/Si substrate, respectively. The results are very distinct when compared to data for single-PANI nanowires. Clear peaks in the SMNWs for the element Zn as compared to this element in the single-PANI nanowires are visible. Other evidence for ZnO NP entrapment is the increased oxygen peaks in the SMNW as compared to images of the single PANI. The different ZnO NP concentration and specific distribution of ZnO NPs in the SMNWs might have resulted in different Zn and O intensities in the EDX scanning data.

The Raman spectrum of Figure 2b shows that the fabricated single nanowire is materialized with doped PANI presenting the peaks on 1,590 cm^-1 ^(C = C bonding), 1,480 cm^-1 ^(C = N bonding), 1,431 cm^-1 ^(C-C stretching), 1,220 cm^-1 ^(C-N stretching), 1,165 cm^-1 ^(in-plane C-H bending), 840 cm^-1 ^(amine deform), 779 cm^-1 ^(ring deform), and 750 cm^-1 ^(imine deform) [25]. Therefore, it is clear that our electrochemical growth method works to electro-polymerize aniline for fabrication of SMNW.

**Figure 3:**
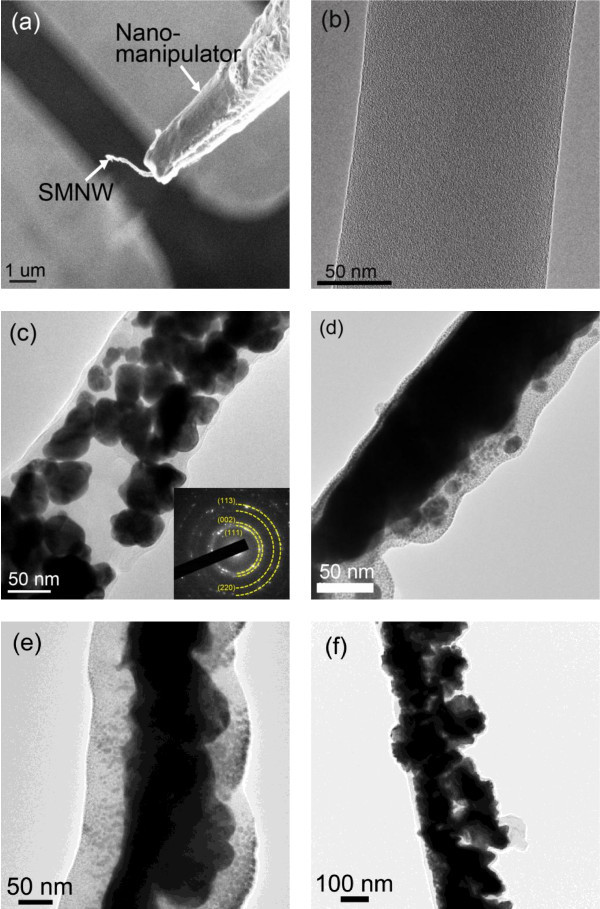
**HRTEM images of single PANI nanowire and SMNWs with different ZnO NPs concentrations**. (a) Single 10 wt.% ZnO NPs-entrapped nanowire was extracted from the electrode and transferred to the TEM sample grid by a nanomanipulator. The SMNW was scratched laterally at the end of nanowire and detached. **(b) **HRTEM image of single PANI nanowire: 0 wt.% ZnO NP concentration. ZnO NPs are absent in this single nanowire. Note the good width uniformity of 137.5 nm. **(c) **HRTEM image of 5 wt.% ZnO NPs (width, 154 nm) and **(d) **10 wt. % ZnO NPs-entrapped PANI nanowires (width, 113 nm). The diffraction pattern for each SMNW is shown in the corresponding inset. Note the ring patterns (PANI) and dots indicating randomly oriented crystalline structure (ZnO). **(e) **1 wt.% ZnO NPs-entrapped PANI nanowire from applying a current of 900 nA in the growing condition. **(f) **2.5 wt.% ZnO NPs-entrapped PANI nanowire from applying a current of 900 nA in the growing condition. The SMNWs of (e) and (f) show tightly agglomerated ZnO NPs inside the nanowire similar to the nanowire of (d).

For more in-depth examination of ZnO NP entrapment, a TEM was utilized. A high-resolution transmission microscopy (HRTEM) was utilized to find the diffraction pattern of individual nanowires. Figure 3a shows a 10 wt.% ZnO NP-entrapped PANI nanowire between electrodes lifted off prior to placement on a TEM grid using an focused icon beam (FIB) and a nanomanipulator. Other HRTEM images for 0, 5, and 10 wt.% ZnO NP-entrapped PANI nanowires are shown in Figure 3b, c, and 3d, respectively. Additionally, the SMNWs (1 and 2.5 wt.% ZnO NP concentration) created under different growing conditions are demonstrated in Figure 3e, f. From these HRTEM images, entrapped ZnO NPs approximately 30 to 60 nm in diameter can be observed, while the reference single-PANI nanowire (0 wt.% ZnO NP) shows a very uniform structure and a 137.5 nm width with no ZnO NPs in Figure 3b.

For the SMNW, a few interesting effects can be observed. Firstly, the ZnO NPs are distributed almost randomly and display a degree of aggregation as shown in Figure 3c and 3d. The comparison between the two different ZnO NP concentrations shows increased agglomeration of the 10 wt.% when compared with the 5 wt.%. From these images, we can see that in low ZnO NP concentrations, entrapped ZnO NPs disperse almost randomly within the nanowire core structure during the in situ polymerization process, with signs of agglomeration occurring. As the ZnO NP concentration increased, the ZnO NPs formed a continuous structure inside of the SMNW - similar to an amorphous ZnO nanowire as shown in Figure 3d. The 10 wt.% SMNW shows the point at which saturation of ZnO NP entrapment occurs in the SMNW structure. Images taken for SMNW with concentrations higher than 10 wt.% showed little difference in the nanowire morphology as a result of saturation at 10 wt.%. The insets of Figure 3c display the ring diffraction patterns for each SMNW. These diffraction patterns can be attributed to the random orientation of the ZnO NPs as well as the polycrystalline structure of PANI. The x-ray diffraction pattern of the entrapped ZnO grown via electrochemical deposition was observed at room temperature. The observed diffraction patterns were (113), (002), (111), and (220) in all directions as shown in Figure 3c.

Secondly, the entrapment of ZnO NPs in the SMNW is also dependent on the amount of current used in the fabrication of the nanowire (See Figure 3e, f). The SMNWs of the 1 and 2.5 wt.% ZnO NP were fabricated in the condition 900 nA current, which is a higher current than the current (600 nA) applied to the SMNWs in Figure 3c and 3d. The higher applied current in fabrication of the nanowire induced a higher electric field inside the nanochannel and attracted more ZnO NPs into the nanochannel. As shown in the HRTEM images Figure 3e and 3f, for the SMNWs, the different growing condition of high applied current shows the feasibility of tuning entrapment of ZnO NPs in low concentrations of ZnO NPs (1 and 2.5 wt.%).

### Characterization of fabricated single multicomposite nanowires

**Figure 4:**
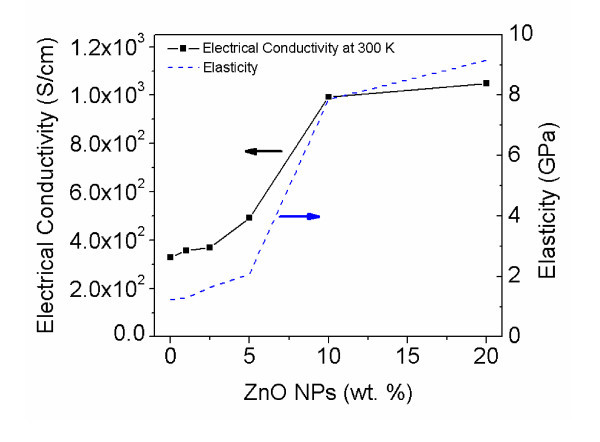
**Enhancement of physical properties of the SMNWs**. Electrical conductivity (solid line at 300 K) and elasticity (dash and double dot line) are measured for 0, 1, 2.5, 5, 10, and 20 wt.%. Note the dramatic increase in each from 2.5 to 10 wt.% ZnO NP concentration. The slopes of physical properties decrease after 10 wt.%, resulting from ZnO NP saturation.

Post-fabrication, the electrical and mechanical properties of SMNWs fabricated in the same growing condition (600 nA current) were characterized. First, the I-V measurements of SMNWs with various concentrations were taken and compared with the measurements of single-PANI nanowires. Figure 4 displays the results when plotting electrical conductivities as a function of the ZnO NP concentration. Electrical conductivities at 300 K were calculated from the I-V curves and the dimensions of the nanowire (from SEM and AFM measurements). The SMNW clearly shows increased electrical conductivity compared with the single-PANI nanowire. The single-PANI nanowire electrical conductivity is 3.30 ± 0.03 × 10^2 ^S cm^-1^. The ZnO NPs-entrapped PANI nanowires' conductivity (300 K), on the other hand, varied from 3.58 ± 0.03 × 10^2 ^to 1.05 ± 0.21 × 10^2 ^S cm^-1 ^in Figure 4. Electrical conductivity demonstrated increases linearly as the ZnO NP concentration increased. In contrast, the increase of electrical conductivity slowed down in the range of concentrations higher than 10 wt.%, with the data trend showing logarithmically increasing behavior.

To study the load strength of the nanowires, the elasticity was measured taking an AFM Force-Displacement (FD) measurement. The insulating layer below the nanowire was first removed using a buffered oxide etchant. For the FD measurement, the deflection of the nanowires was obtained by pushing down and up at the center of the nanowires with a load of 5 nN. For the calculations, the free-standing nanowire was assumed to be a beam supported at both ends. The deflection of the nanowire was measured from the FD measurement curve. Young's modulus of the nanowires was then calculated using the deflection of nanowire and applied force by the AFM.

Figure 4 shows the results of such calculations. Young's modulus of the SMNW is distinctly larger than that of the single PANI nanowire. The elasticity of the single-PANI nanowire ranged from 1.24 to 3.46 GPa depending on the shape of the nanowire - in keeping with the 2 to 3-GPa elasticity of PANI microfiber found in previous studies [26]. From 1 to 5 wt.% ZnO NPs concentrations, the ZnO NPs-entrapped PANI nanowires have a Young's modulus measurement similar to the single-PANI nanowire, with a modulus of 1.3 and 2.1-GPa, respectively. This is attributed to the dominance of the PANI in terms of volume of the nanowire, since those concentrations do not form a continuous link that could increase stiffness of the nanowire. Subsequent measurements of SMNWs with 10 and 20 wt.% have a modulus estimated to be 7 and 9 GPa, respectively as shown in Figure 4. The limited increase here is caused by the saturation of ZnO NP. Although much lower than quoted values of the ZnO nanowire Young's modulus [27], it should be noted that the SMNW contains only entrapped ZnO NPs and its elasticity is not comparable to single-crystal ZnO nanowire measurements. When all the results are plotted, Young's modulus changes logarithmically with ZnO NP concentration. We suggest that this improvement of elasticity in the SMNWs is caused by the reaction between PANI and ZnO NPs from in situ polymerization [28,29]. The elasticity of SMNW shows a saturation behavior similar to the electrical conductivity in high ZnO NP concentration of 10 wt.%.

## Discussion

The enhanced electrical conductivity may be the result of various mechanisms. It could be a result of a structural change in the SMNW and the reaction between the ZnO NP and the PANI as noted elsewhere [17,28,29]. In pure single-PANI nanowires, electrical conductivity is defined by electron transfer along the backbone of PANI [26,30,31]. On the other hand, the SMNW may provide multiple electron pathways through both the PANI and ZnO NPs for increased conductivity [32]. The internal structure of the fabricated SMNWs observed through use of the HRTEM indicates that the single-PANI nanowire and the SMNWs have different internal ZnO NP arrangements. Qualitatively, by comparing the fraction of entrapped ZnO NPs, PANI would be a dominant conducting material for below 5 wt. %. Alternatively, we hypothesize that a continuous ZnO NP structure in over 10 wt. % may be dominant for an electron transfer pathway. The presence of this continuous ZnO structure explains why the increase of conductivities begins to slow down at certain concentrations. In the saturation of ZnO NP in the SMNWs, the continuous structure of ZnO NPs as shown in Figure 3d, like ZnO nanowire, provides an electron pathway for electrons to move about freely in the SMNW [30,32]. The improvement of electron transfer in the nano-multicomposite thin films via in situ polymerization of PANI with ZnO NPs has also been previously reported [28,29,32]. In addition, the saturation behavior in regard to electrical conductivity is well known in macro- and micro-multicomposite materials [32,33]. Between 5 and 10 wt.%, we can only surmise that the mechanism of electron transfer consists of a mix of both, which are dominant electron transfers in PANI or continuous ZnO structure, indicating a strong dependence on the random placement of the ZnO NP during growth.

For the fabricated SMNWs, we assume the dominant mechanism of electrical conductivity is a mixture of hopping and tunneling, depending on the different structure of ZnO NP entrapment in Figures 3 and 4. The dispersed ZnO NPs are spaced less than 10 nm apart, indicating that tunneling may be dominant - especially at higher applied electric fields as shown in Figure 3c. For low ZnO NP concentration below 5 wt.%, Poole-Frenkel emission or another hopping mechanism in PANI may be superior due to the random distribution of ZnO NPs [34]. We have investigated evidences for hoping conduction in the SMNW using Mott-Davis model [35]. Our calculation indicates the temperature dependence of the conductivity for the fabricated SMNWs. Therefore, the number of possible hoping sites available with temperature may enhance electrical conductivity [36,37].

## Conclusions

SMNW with ZnO NPs, a new novel material, was fabricated using an electrochemical growth method. This electrochemical growth method is an easy and effective method to fabricate site-specific, uniform, and reproducible nanowires bridged between two electrodes. The entrapment of ZnO NP inside the nanowire was validated by use of an SEM, EDX, and TEM. Additionally, when the electrical conductivity and elasticity of the SMNWs were varied in a logarithmic fashion by varying the ZnO NP concentration in the electrochemical growth aniline solution, variations in electrical conductivity and elasticity of SMNW displayed saturation behavior in accordance with the ZnO NPs concentration.

The HRTEM images and characterization revealed different NP entrapment inside the SMNW and different effects of ZnO NP concentration on its physical properties. Beyond 10 wt.%, the ZnO NP entrapment resulted in hardly any change in physical properties. Note, however, that we suggest a logarithmic relationship for the concentration of the growth solution and not the concentration inside the SMNW - a very stark difference. The nature of this relationship might have to do with some activation energy for NPs successfully polymerizing into the nanowire during growth. From our results, it seems that the appropriate ZnO NP concentration, between 5 and 10 wt.%, provides regularly dispersed entrapment of functionalized ZnO NPs in the SMNW.

Based on the advantages of PANI and ZnO NPs, such as good bioaffinity and electrical conductivity which can be controlled according to growth condition such as ZnO NP concentration, we suggest that these SMNWs can be successfully employed as advanced biosensing materials [38]. This method of SMNW fabrication is easily applicable for biosensor or electrical devices with controllable and enhanced properties.

## Methods

The nanochannel and the pre-patterned electrodes were built up lithographically using an e-beam evaporator (VE-180, Thermionics, Hayward, CA, USA) and an electron beam lithography machine (e-LiNe, Raith, Dortmund, Germany), explained in detail elsewhere [18-20]. Aniline solutions utilized in the nanowire growth process (0.01 M aniline in 0.1 M HCl) with ZnO NPs (1, 2.5, 5, 10, and 20 wt.%) were sonicated for 60 to 90 min to disperse NPs homogeneously in the solution. Aniline and ZnO NPs were obtained from Sigma-Aldrich and Alfa Aesar, respectively. During sonication, the temperature of the solution was kept below 50°C to prevent high-temperature agglomeration of the NPs.

After sonication, nanowire growth was achieved using a probe station and a semiconductor device analyzer (B1500A, Agilent); a 0.4-μ L solution was dropped over the nanochannel while a static current of 600 or 900 nA was applied between the two metal electrodes. The measured voltage between the two metal electrodes was monitored via a semiconductor device analyzer. Growth of the nanowire was completed when the voltage across the nanowire dropped to the order of microvolts, indicating a short circuit had been achieved [18-20]. Post-growth, the SMNWs were immersed in acetone for 2 min and rinsed in deionized water for 1 min.

The morphology of the SMNWs was studied using SEM (e-LiNE, Raith, Dortmund, Germany) and an AFM (XE-100, Park Systems, Suwon, S. Korea) in non-contact mode. EDX (XL-30, Philips, Eindhoven, The Netherlands) was utilized to reveal the elemental composition of the nanowires and validate our claim of ZnO NP entrapment in the nanowires. In order to verify ZnO NP entrapment in a single nanowire, HRTEM (JEM-2100F, JEOL, Tokyo, Japan) images were obtained, with SMNW samples extracted by etching with a Focused Ion Beam (FIB, Nova 200 NanoLab, FEI Company, Hilsboro, OR, USA) and a nanomanipulator (F100 Nanomanipulator, Zyvex, Richardson, TX, USA). In this process, the SMNWs were detached from the two electrodes by laterally scratching the surface and were then transferred to a TEM grid using the nanomanipulator. HRTEM was carried out at an acceleration voltage of 200 kV and a camera constant of 25 cm. HRTEM was utilized to confirm entrapment and examine alignment and distribution of the ZnO NPs inside the SMNWs. Firmly, Raman Spectroscopy (inVia, Renishaw, Wotton-under-Edge, UK) confirmed that the fabricated nanowires are materialized through electropolymerization as doped PANI in Figure 2b. Physical properties of the SMNWs were measured with I-V curves and deflection of the nanowire using a semiconductor device analyzer and FD measurements obtained from an AFM, respectively. Electrical conductivity was calculated from the measured I-V curve along with dimensions of the nanowire. The applied force of 5 nN used in the AFM FD measurements was performed at the center of the nanowire, with both ends supported.

## Competing interests

The authors declare that they have no competing interests.

## Authors' contributions

IL carried out the fabrication of SMNWs, SEM characterization, EDX measurement, AFM measurement, I-V measurement, and AFM FD measurement. SYP and MJK carried out TEM characterization. MY conceived of the study, and participated in its design and coordination. All authors read and approved the final manuscript.

## Abbreviations

AAO: anodic aluminum oxide; AFM: atomic force microscope; BOE: buffered oxide etchant; CNT: carbon nanotube; EDX: energy-dispersive x-ray spectroscopy; FD: force-displacement; FIB: focused ion beam; HRTEM: high-resolution transmission microscopy; NP: nanoparticle; PANI: polyaniline; Ppy: polypyrrole; SEM: scanning electron microscopy; SMNW: single multicomposite nanowire; VRH: variable hopping model.
